# Evaluation of Cx43 Gap Junction Inhibitors Using a Quantitative Structure-Activity Relationship Model

**DOI:** 10.3390/biomedicines11071972

**Published:** 2023-07-12

**Authors:** Ramona Matusevičiūtė, Eglė Ignatavičiūtė, Rokas Mickus, Sergio Bordel, Vytenis Arvydas Skeberdis, Vytautas Raškevičius

**Affiliations:** 1 Faculty of Medicine, Lithuanian University of Health Sciences, 03101 Kaunas, Lithuania; ramona.matuseviciute@stud.lsmu.lt (R.M.); egle.ignataviciute@stud.lsmu.lt (E.I.); 2 Institute of Cardiology, Lithuanian University of Health Sciences, 50162 Kaunas, Lithuania; rokas.mickus@lsmu.lt (R.M.); sergio.bordel@lsmu.lt (S.B.); arvydas.skeberdis@lsmu.lt (V.A.S.); 3 Institute of Sustainable Processes, University of Valladolid, 47011 Valladolid, Spain

**Keywords:** Cx43, gap junctions, conductance, inhibitors, docking, IC_50_

## Abstract

Gap junctions (GJs) made of connexin-43 (Cx43) are necessary for the conduction of electrical impulses in the heart. Modulation of Cx43 GJ activity may be beneficial in the treatment of cardiac arrhythmias and other dysfunctions. The search for novel GJ-modulating agents using molecular docking allows for the accurate prediction of binding affinities of ligands, which, unfortunately, often poorly correlate with their potencies. The objective of this study was to demonstrate that a Quantitative Structure-Activity Relationship (QSAR) model could be used for more precise identification of potent Cx43 GJ inhibitors. Using molecular docking, QSAR, and 3D-QSAR, we evaluated 16 known Cx43 GJ inhibitors, suggested the monocyclic monoterpene d-limonene as a putative Cx43 inhibitor, and tested it experimentally in HeLa cells expressing exogenous Cx43. The predicted concentrations required to produce 50% of the maximal effect (IC_50_) for each of these compounds were compared with those determined experimentally (*p*IC_50_ and *e*IC_50,_ respectively). The *p*IC_50_ies of d-limonene and other Cx43 GJ inhibitors examined by our QSAR and 3D-QSAR models showed a good correlation with their *e*IC_50_ies (R = 0.88 and 0.90, respectively) in contrast to *p*IC_50_ies obtained from molecular docking (R = 0.78). However, molecular docking suggests that inhibitor potency may depend on their docking conformation on Cx43. Searching for new potent, selective, and specific inhibitors of GJ channels, we propose to perform the primary screening of new putative compounds using the QSAR model, followed by the validation of the most suitable candidates by patch-clamp techniques.

## 1. Introduction

Gap junctions (GJs) are intercellular channels indispensable for electrical interaction between cardiac myocytes and synchronized cardiac contraction [1]. Connexin (Cx)-based GJ channels are formed of two opposing hemichannels in contiguous cells (reviewed in [2]). Six Cx subunits compose a hemichannel with an ion-selective pore. Each Cx protein has four alpha-helical transmembrane domains (TMs), intracellular N- and C-termini, two extracellular loops, and a cytoplasmic loop. The family of Cx genes consists of 21 members in the human genome. The prevailing connexin isoform in human cardiac tissue is Cx43 [3]. Changes in the function, expression, or localization of Cx43 are associated with a higher frequency and severity of arrhythmias and sudden death in patients with cardiovascular diseases [3]. On the other hand, modulation of GJ function is onerous due to the shortage of specific and Cx-type-selective GJ inhibitors. Most GJ inhibitors are non-specific compounds, such as antimalarial drugs, polyamines, glycyrrhetinic acid, volatile anesthetics, arachidonic acid, cyclodextrins, anti-cancer drugs cisplatin and oxaliplatin, fatty acid amides, terpenes [4,5,6], and other drugs. In addition, most of these compounds have been shown to inhibit not only intercellular communication through GJs but also the membranous Na^+^, K^+^, and Ca^2+^ channels that are crucial for the generation and spread of action potential [7,8,9].

Molecular docking approaches predict the binding affinities of compounds relatively accurately; however, high-affinity compounds are not necessarily potent inhibitors [10,11]. Quantitative structure-activity relationship (QSAR) is an in silico modeling method used for determining binding affinities and biological activities of compounds from their structural features [12]. QSAR analysis is particularly useful in the pharmaceutical industry, as this method helps to select substances according to their desirable biological activity, thus significantly reducing the number of substances that need to be tested in vitro and in vivo [12]. QSAR outperforms molecular docking methods, which, in the absence of a reliable 3D receptor-ligand complex structure, face limitations [13]. Modulation of Cx43 GJ activity (activation or inhibition) is expected to be beneficial in the management of cardiac pathologies [14], such as ischemic heart disease, heart failure, hypertrophic cardiomyopathy, arrhythmogenic right ventricular cardiomyopathy, and others [15]. Therefore, the aim of this study is to evaluate known and putative Cx43 inhibitors using molecular docking, QSAR, and 3D-QSAR, and compare predicted IC_50_ (*p*IC_50_) values with experimental ones (*e*IC_50_).

## 2. Materials and Methods

### 2.1. Bioinformatic Analysis

Open-access databases were searched for studies on the inhibitory effects of various substances on Cx43 GJs. The following compounds, belonging to different classes and with their *e*IC_50_ies, were found [4,14,15,16,17,18,19,20,21]: 2-aminoethoxydiphenyl borate (2-APB, organoboron compound); α-copaene, α-pinene, and sabinene (terpenes); digoxin (glycoside); dihydrogambogic acid (DGBA, pyranoxanthone); heptanol (fatty alcohol); flufenamic acid (FFA); and meclofenamic acid (MFA) (anthranilic acid derivatives); mefloquine and quinidine (quinolines); dicumarol and warfarin (anticoagulants); 18α-glycyrrhetinic acid (18α-GA); 18β-glycyrrhetinic acid (18β-GA); and carbenoxolone (CBNX) (triterpenoids). In such a way, the dataset for Cx43 inhibition modeling was made up of 17 compounds, including monoterpene d-limonene [22,23,24] that was later suggested by molecular modeling as a putative GJ inhibitor. D-limonene was selected due to its structural similarity to other terpenes (sabinene, α-pinene, and α-copaene), which we have recently identified as new Cx43 GJ inhibitors [4]. For the sake of clarity, the PubChem CID of each investigated inhibitor is also provided.

### 2.2. Molecular Docking

The structure of human Cx43 (hCx43) (PDB ID 7F94) [25] was used for molecular docking, and the I164V mutation was introduced with ChimeraX software (v. 1.6.1) to represent the rat Cx43 (rCx43) structure. In total, there are seven single-point variations between hCx43 and rCx43, but six of them are either in the intracellular loop or in the C-terminal domain, which are not provided in the hCx43 structure [25]. The resulting rCx43 model was validated with ProSA [26] and Procheck (v. 3.5.4) [27]. Three-dimensional molecular structures of ligands were obtained from the PubChem database [28]. Ligands without clear 3D structures (digoxin and DGBA) or organoboron compounds (2-APB) (no modeling parameters for the boron atom present in 2-APB) were excluded from docking. Smina (v. Oct 15 2019) docking software [29] with a customized Vina scoring function [30] was used. In order to prevent symmetric docking conformations into neighboring subunits, the docking mode was configured to cover two adjacent Cx43 hemichannel subunits (precise coordinates (x, y, z) were center 118; 160; 95; and size 43; 40; 90). The random seed was always set to 1. Depending on the referred species (Table 1), the respective ligand was docked into the rCx43 or hCx43 model. All ligand output conformations except the top-scoring ones were automatically excluded from further analysis with program settings, leaving only the most reliable one for each ligand. Three-dimensional docking images were generated with ChimeraX software [31], and 2D docking plots were generated with LigPlot+ (v. 2.2) software [32]. LigPlot+ molecular interaction parameters were kept as default (2.90 Å minimal and 3.90 Å maximal contact distances for all non-bonded contacts). Log(*p*IC_50_) from molecular docking was calculated using the equation of linear regression:

(1) $$-\log\left(pIC_{50}\right)=m\cdot \mathrm{DMA}+n\;$$

where *m* and *n* are fitting coefficients, and DMA is Docking Minimized Affinity. When DMA is equal to 0, the intercept (*n*) is −log(*p*IC_50_). The slope (*m*) determines the direction and rate of −log(*p*IC_50_) change when DMA increases [33].

### 2.3. Development of the QSAR Model

QSAR model development requires molecular descriptors calculated from their chemical structure. PaDEL descriptors [34] and ChemoPy descriptors [35] were calculated using the ChemDes web-based platform [36]. In total, 3010 molecular descriptors were calculated for the investigated compounds. Descriptors having identical values for all analyzed compounds were considered useless and were excluded. In total, 1478 molecular descriptors remained for further modeling.

Regression analysis methods are statistical instruments widely used for the determination of relationships between molecular descriptors and the biological activities of compounds [37]. Multiple linear regressions (MLR) [38,39] for QSAR were developed using R software (v. 4.3.0) with a Leaps package. Our QSAR model describes the predicted biological activities of compounds by following the MLR:

(2) $$-\log\left(pIC_{50}\right)=a_{1}x_{1}+a_{2}x_{2}{+}_{\ldots }+a_{n}x_{n}+b$$

where *p*IC_50_ is the predicted concentration required to produce 50% of the maximal effect; *x_n_* is a molecular descriptor calculated by the software; *a_n_* is its fitting coefficient; and *b* is the −log(*p*IC_50_) value when all molecular descriptors are equal to 0. Given the limited size of the data set, the number of chosen descriptors (*n*) for the final model was limited to three to avoid overfitting [40]. All possible models with three descriptors were created and analyzed, searching for the one providing the most significant correlation between *p*IC_50_ and *e*IC_50_ values. Promising QSAR models were transferred into Microsoft Excel (v. 2019) sheets for final inspection and validation. Microsoft Excel Analysis ToolPak was used to compute the final values of the regression analysis: R—coefficient of correlation—measured both the strength and the direction of a linear relationship; R^2^—coefficient of determination—provided the percentage variation, making it easier to compare between different models; adjusted R^2^ helped identify problems with overfitting.

### 2.4. Modeling in 3D-QSAR

More than 35 years ago, 3D-QSAR was introduced to find statistical correlations between molecular interaction fields (MIFs) and biological activity useful for rationalizing existing data and making further predictions [41]. For 3D-QSAR modeling, the same set of Cx43 inhibitors as for molecular docking was used. Before performing 3D-QSAR, the alignment of molecular structures was carried out using OPEN3DALIGN (v. 2.3) [42], which created 14 alignments using each Cx43 inhibitor as a template; each alignment received a score of O3A_SCORE. The molecular alignment file with the highest O3A_SCORE was adjusted by adding the available experimental inhibition values of compounds. OPEN3DQSAR (v. 2.3) was used to perform 3D-QSAR [43]. A grid box set with a 1.0 Å step size and a 5.0 Å output gap was used to calculate the Van der Waals and electrostatic MIFs. The extreme values of MIFs were removed according to the cutoff values given in the software manual. Data from these refined MIFs were used to carry out the partial least squares (PLS) regression for correlation with the −log(*p*IC_50_). Open-Source PyMOL (v. 2.6.0) was used to visualize the final PLS coefficient color maps and to make 3D-QSAR molecular images [44]. For the final images, a 0.0002 (for the positive contribution) or −0.0002 (for the negative contribution) molecular field cutoff was used. Molecular field cutoffs are necessary to generate final images since, without them, the investigated molecular fields would cover the entire investigated space.

### 2.5. Cell Lines and Culture Conditions

Experiments were performed on HeLa (human cervix carcinoma, ATCC CCL-2, Manassas, VA, USA) cells stably transfected with rCx43 tagged with a green fluorescent protein (Cx43-EGFP). A stable HeLa cell line expressing Cx43-EGFP was obtained in collaboration with Dr. F. Bukauskas (Albert Einstein College of Medicine, New York, NY, USA). The construction protocol of the vector is described elsewhere [45]. A cell line expressing Cx43-EGFP was selected using 500 µg/mL G418/geneticin (Sigma-Aldrich Co., Saint Louis, MO, USA). Cells were grown in DMEM medium containing 10% fetal bovine serum (FBS), and a penicillin/streptomycin mix (100 U/mL penicillin and 100 μg/mL streptomycin; Gibco Laboratories) at 37 °C and 5% CO_2_. Typically, the cells were analyzed on the second day after passage.

### 2.6. Electrophysiological Measurements

For electrophysiological recordings, the cells grown on glass coverslips were transferred to an experimental chamber with constant flow-through perfusion, mounted on the stage of the inverted microscope Olympus IX81 equipped with the Orca-R^2^ cooled digital camera. Junctional conductance g_j_ between contiguous cells was measured using the dual whole-cell patch-clamp technique [46]. Cell-1 and Cell-2 of a cell pair were voltage clamped independently with the patch-clamp amplifier MultiClamp 700B (Molecular Devices, Inc., San Jose, CA, USA) at the same holding potential, V_1_ = V_2_. By applying a repetitive voltage ramp every 10 s (−10 mV, 20 ms) in the Cell-1 (ΔV_1_) and keeping the other constant, the junctional current was measured as the change in current in the Cell-2, I_j_ = ΔI_2_. Thus, g_j_ was obtained from the ratio −I_j_/ΔV_1_, where ΔV_1_ is equal to transjunctional voltage (V_j_), and a negative sign indicates that the junctional current measured in Cell-2 is oppositely oriented to the one measured in Cell-1. Voltages and currents were digitized using the Digidata 1440A data acquisition system (Molecular Devices, Inc., San Jose, CA, USA) and acquired and analyzed using pClamp (v. 10) software (Molecular Devices, Inc., San Jose, CA, USA). The filtering frequency was 4 kHz, and the sampling rate was 2 kHz. Patch pipettes, their filling solution, and a modified Krebs-Ringer solution for cell perfusion were prepared as described previously [4]. All chemical reagents were purchased from Sigma-Aldrich Corp. Stock solutions of d-limonene were prepared in dimethyl sulfoxide at a 100 mM concentration and later diluted with modified Krebs-Ringer solutions to the necessary concentration (10, 30, 50, or 100 µM).

### 2.7. Statistical Analysis

The dose–response curve obtained with different concentrations of d-limonene was fitted to a three-parameter Hill’s equation, and the concentration of the compound required to produce 50% of the maximal effect (IC_50_) was derived using the SigmaPlot (v. 12.0, Systat Software, Inc., Erkrath, Germany) software. Data are reported as means ± SEM.

## 3. Results

### 3.1. Molecular Docking of Cx43 Inhibitors

Upon rCx43 model validation, it was found that the z-score of the model (−2.98) is within the range of scores typically found for native proteins of similar size. According to the PROCHECK Ramachandran plot analysis, a majority of the residues (89.3%) are located in the core region, with 10.7% in the allowed region and 0.0% in the generously allowed region. Notably, no residues were detected in the disallowed region, indicating that all residues have an acceptable conformation. The Goodness factors (G-factors) from the PROCHECK results indicate the quality of covalent and overall bond/angle distances. Specifically, the dihedral G-factor was observed to be −0.20, while the covalent and overall G-factors were 0.51 and 0.09, respectively, in the present model. The C-terminal domain and intracellular loop are not included in the final model, as they are not provided in the experimental structure either [25]. Such a model was used for further molecular docking simulations.

Further optimizing the Vina scoring function for the membrane protein Cx43, different hydrophobic interaction weights were applied in a range from 0 to −2 with a step of 0.1, and finally, the optimal hydrophobic interaction weight was set to −0.2 for the molecular docking calculations.

**Figure 1:**
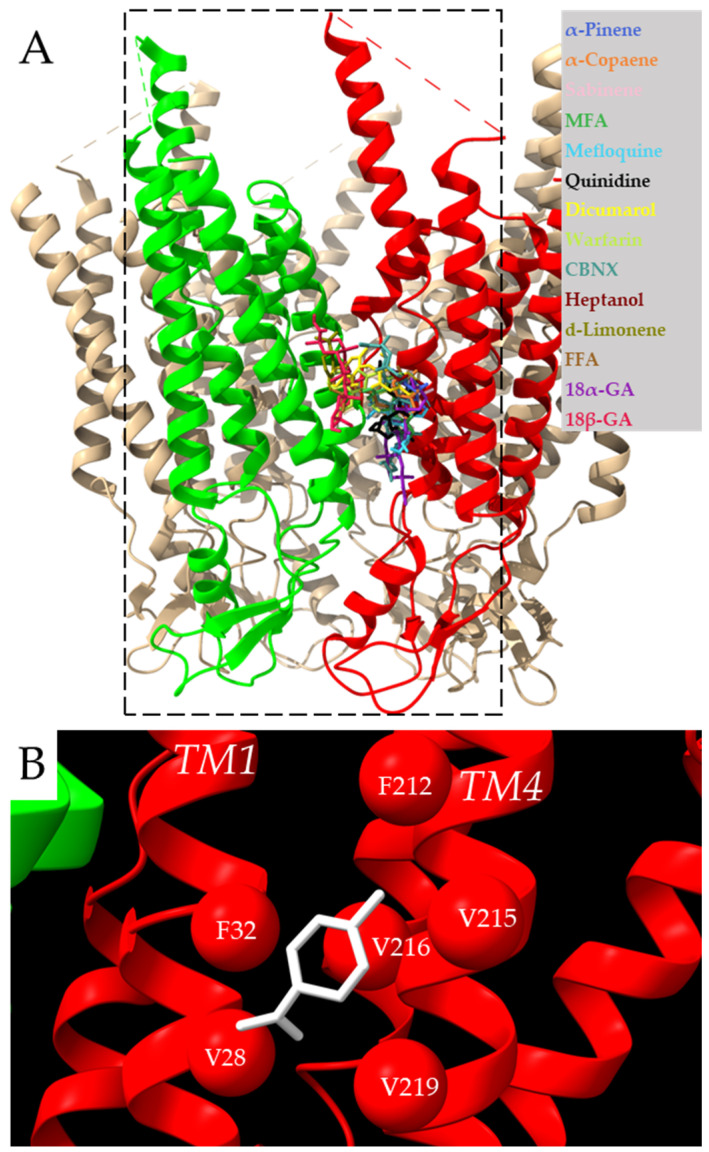
Docking of examined compounds to the Cx43 GJ channel. (**A**) Cx43 hemichannel with highlighted in red and green two adjacent subunits docking the examined compounds (indicated in different colors) on the single subunit or between neighboring subunits (see Figure 2 for details). The dotted rectangle represents the docking box covering two neighboring Cx43 subunits with the exact coordinates indicated in the methods section. Intracellular loops missing in the model are marked with dotted lines. (**B**) Molecular docking conformation of d-limonene. Different interacting Cx43 transmembrane domains are marked as TM1 and TM4 helixes, and amino acid residues interacting with d-limonene (purple) are marked as balls.

Molecular docking showed a common docking site for all examined compounds. This common docking site is a large hydrophobic furrow between the TMs of neighboring Cx43 subunits (Figure 1A, Supplementary Video S1 and Supplementary Figure S1). These results suggest that compounds of a highly hydrophobic nature and appropriate size could be tested as putative Cx43 inhibitors. Therefore, the monoterpene d-limonene, which has all these properties, was chosen as a possible Cx43 inhibitor.

**Figure 2:**
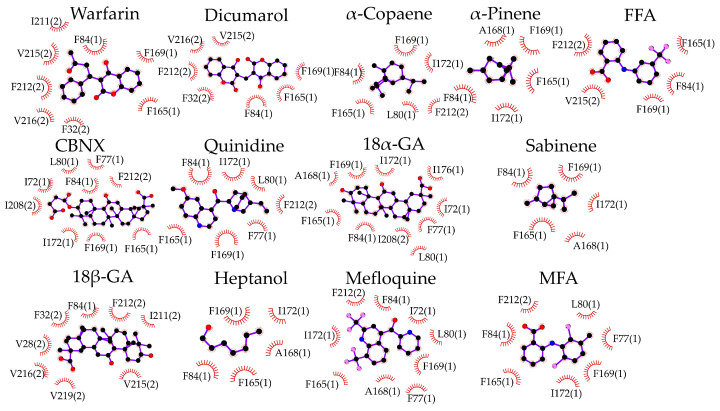
Specification of molecular docking of examined compounds to the Cx43 GJ hemichannel. Interacting amino acids are presented with their number in the Cx43 sequence and the Cx43 subunit number in parenthesis. Hydrophobic interactions are shown as brick-red spoked arcs; black balls indicate carbon atoms; red—oxygen; blue—nitrogen; and pink—halogen.

The docking of d-limonene (Figure 1B) shares the same site with the other compounds. The amino acids interacting with the investigated inhibitors are specified in 2D plots (Figure 2). It is necessary to note that F84, F165, and F169 are highly common residues often found at the exact docking site of these compounds.

**Figure 3:**
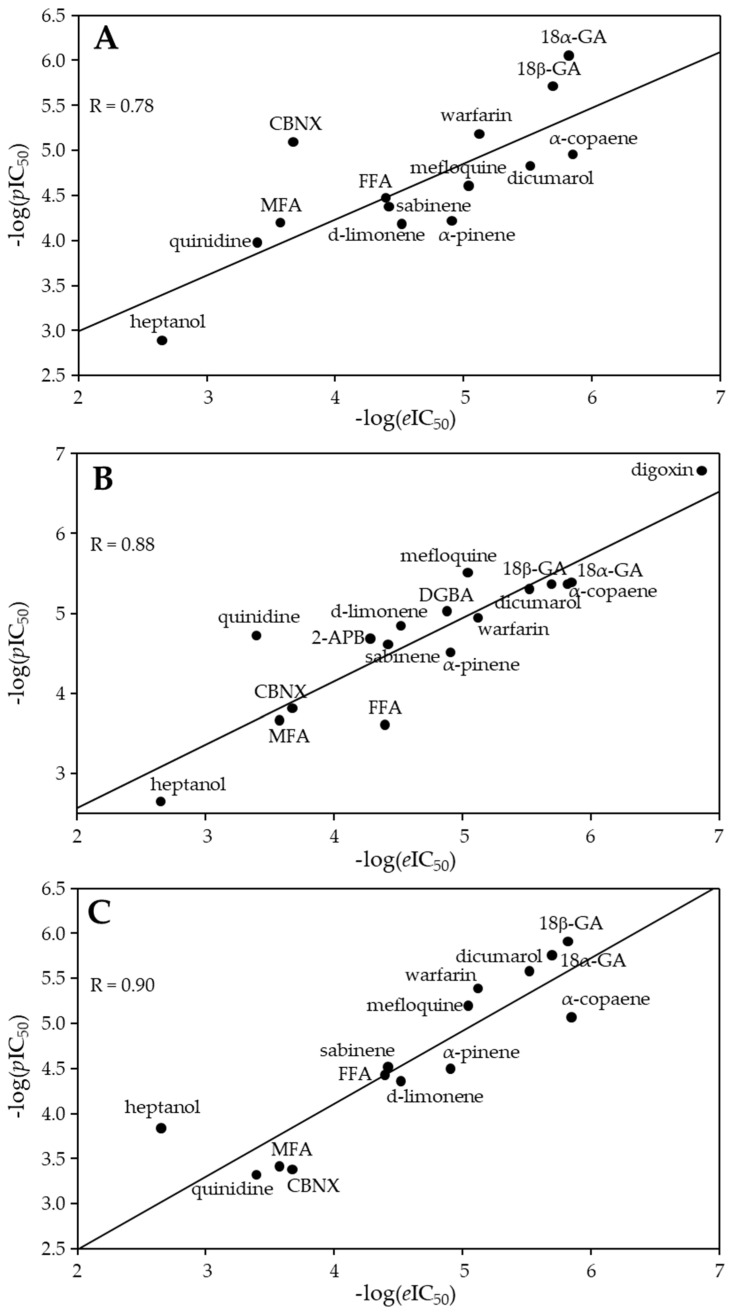
The plot of −log(*e*IC_50_) values versus −log(*p*IC_50_) of the docked Cx43 inhibitors (**A**). The plot of −log(*e*IC_50_) values versus −log(*p*IC_50_) values from the QSAR model (**B**) and the 3D-QSAR model (**C**) for the Cx43 inhibitors.

From linear regression (Equation (1)), *m* and *n* values were found to be −0.18 and 0.75, respectively. Using these values, the *p*IC_50_ was calculated from docking minimized affinity for each compound (Table 1). A fair correlation (R = 0.78) between *p*IC_50_ies and *e*IC_50_ies was found, presumably due to quite frequent discrepancies between compound affinity and potency (Figure 3A).

### 3.2. QSAR Modeling of Cx43 Inhibitors

Using the R leaps package-based approaches, the molecular descriptors SpMin5_Bhm, SpMax3_Bhi, and minHBd were selected for QSAR modeling of Cx43 inhibition. More detailed information about those descriptors can be found in the PaDEL-descriptor software (v. 2.21) manual [34]. Using them, the following QSAR model was developed for Cx43 inhibition:

(3) $$-\log\left(pIC_{50}\right)=-4.87\times \mathrm{SpMin}5\_\mathrm{Bhm}+5.67\times \mathrm{SpMax}3\_\mathrm{Bhi}-5.70\times \mathrm{minHBd}-7.24\;$$

The developed QSAR model allowed the calculation of the *p*IC_50_ of each compound from two calculated molecular descriptor values and comparing them with their *e*IC_50_ies. The best QSAR model (Equation (3)) achieved a very strong correlation between *p*IC_50_ies and eIC_50_ies: R = 0.88, R^2^ = 0.79, and R^2^_adj_ = 0.77. The values of −log(*p*IC_50_) calculated with this model are provided in Table 1 together with −log(*e*IC_50_ies). The same values were used in Figure 3B, where −log(*p*IC_50_) was plotted against −log(*e*IC_50_).

**Table 1:** Evaluation data of selected Cx43 GJ inhibitors by molecular docking, QSAR, and 3D-QSAR models.

PubChem CID	Name	−Log(*e*IC_50_)	SpMin5_Bhm	SpMax3_Bhi	minHBd	−Log(*p*IC_50_) from Docking	−Log(*p*IC_50_) from QSAR	−Log(*p*IC_50_) from 3D-QSAR	O3A_SCORE
1598	2-APB [18] (*r*)	4.29 *	1.08	3.51	0.47	-	4.68	-	-
3371	FFA [19] (*r*)	4.40 *	1.13	3.36	0.48	4.47	3.60	4.42	1177
4037	MFA [16] (*h*)	3.58 ^#^	1.14	3.23	0.33	4.19	3.66	3.41	1124
4046	Mefloquine [20] (*h*)	5.05 ^#^	1.28	3.63	0.28	4.60	5.50	5.19	1267
8129	Heptanol [16] (*h*)	2.66 ^#^	0.81	3.11	0.67	2.88	2.64	3.83	598
10114	18β-GA [19] (*h*)	5.70 ^#^	1.63	3.75	0.13	5.71	5.36	5.75	1428
18818	Sabinene [4] (*r*)	4.42 *	1.21	3.13	0.00	4.37	4.60	4.51	701
22311	d-Limonene (*r*)	4.52 *	1.12	3.09	0.00	4.18	4.84	4.35	773
73398	18α-GA [19] (*h*)	5.82 ^#^	1.63	3.75	0.13	6.05	5.36	5.9	1400
441074	Quinidine [20] (*h*)	3.40 ^#^	1.42	3.52	0.20	3.97	4.72	3.31	1136
636403	CBNX [16] (*h*)	3.68 ^#^	1.69	3.76	0.36	5.09	3.81	3.37	1502
2724385	Digoxin [16] (*h*)	6.87 ^#^	1.82	3.84	−0.19	-	6.77	-	-
6857793	DGBA [17] (*h*)	4.88 ^#^	1.80	3.74	0.03	-	5.02	-	-
11240513	α-Pinene [4] (*r*)	4.91 *	1.14	3.05	0.00	4.21	4.51	4.49	719
12303902	α-Copaene [4] (*r*)	5.85 *	1.37	3.40	0.00	4.95	5.38	5.06	890
54676038	Dicumarol [14] (*r*)	5.52 ^#^	1.13	3.63	0.44	4.82	5.30	5.57	1286
54678486	Warfarin [14] (*r*)	5.12 ^#^	1.26	3.61	0.38	5.18	4.94	5.38	1145

*h* and *r* in parenthesis indicate human and rat Cx43, respectively; * patch clamp technique; and ^#^ metabolic communication.

### 3.3. The 3D-QSAR Modeling of Cx43 Inhibitors

The scoring of the alignment based on the structure of each compound (O3A_SCORE) revealed that the carbenoxolone structure was the most useful as a template (Table 1). The best 3D-QSAR model constructed with such a template achieved a very strong correlation between *p*IC_50_ies and *e*IC_50_ies: R = 0.90, R^2^ = 0.81, and R^2^_adj_ = 0.79. On the other hand, the alignment of known Cx43 inhibitors that are quite different in chemical structure was not ideal, and this could lead to impaired 3D-QSAR model robustness. Far higher O3A_Score values can be found in the literature [47]. The 3D-QSAR-calculated −log(*p*IC_50_) values were plotted against −log(*e*IC_50_) and shown in Figure 3C. The values of −log(*p*IC_50_) calculated with this model are provided in Table 1 together with −log(*e*IC_50_).

**Figure 4:**
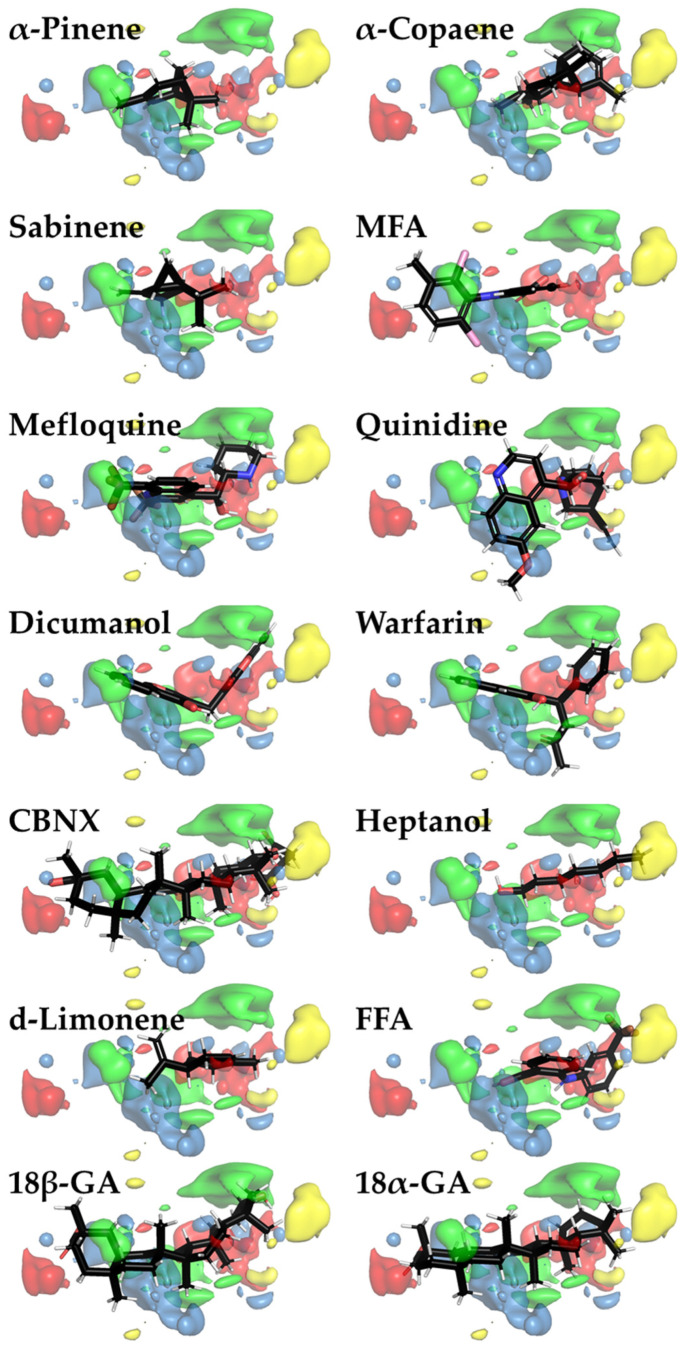
Color maps for 3D-QSAR PLS coefficients for Cx43 inhibitors visualized with PyMOL. The areas where steric bulk has a positive or negative correlation with biological activity are indicated as green or yellow clouds, respectively. Meanwhile, red and blue clouds indicate the regions with positively charged/hydrogen bond donor and negatively charged/hydrogen bond acceptor properties, respectively, that positively correlate with biological activity. Three-dimensional inhibitor structures are depicted as sticks with the respective colors of the chemical element (red—oxygen; black—carbon; white—hydrogen; blue—nitrogen; brown—fluorine; and pink—chlorine).

PLS regression was used to evaluate the correlation between MIFs and −log(*e*IC_50_) of the examined compounds. PLS coefficient color maps around inhibitor 3D structures provide more insights into beneficial and non-beneficial parts of the inhibitor molecules. They are visualized with PyMOL as green (a positive contribution of steric bulk), yellow (a negative contribution of steric bulk), red (a positive contribution of positively charged/hydrogen bond donor), or blue clouds (a positive contribution of negatively charged/hydrogen bond acceptor) (Figure 4). From such images, it can be assumed that a properly sized hydrophobic surface plays the most important role in Cx43 inhibition (Figure 4).

### 3.4. D-Limonene Dose-Dependently Inhibits Cx43 GJ Conductance

**Figure 5:**
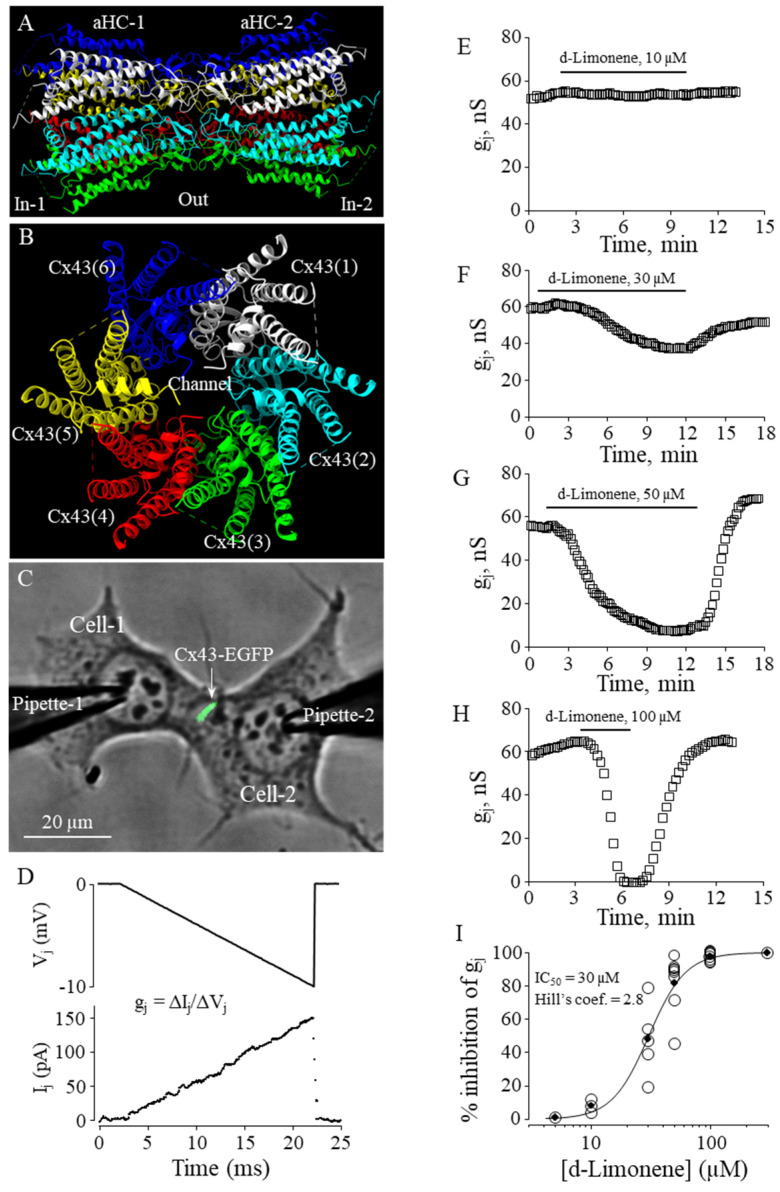
The effect of d-limonene on Cx43 GJ conductance. (**A**,**B**) Cx43 GJ model (side and top views, respectively). (**C**) Dual whole-cell patch-clamp measurement of Cx43-EGFP conductance in HeLa cells. (**D**) G_j_ was measured by applying repeated −10 mV, 20 ms V_j_ ramps, which do not cause the voltage-dependent gating of Cx43 GJ channels that gate at much higher voltages (see, for example, [4]). (**E**–**H**) Typical effects of d-limonene at indicated concentrations on Cx43 GJ conductance. (**I**) Dose-dependence of the d-limonene effect on Cx43 GJ conductance (*e*IC_50_ = 30 µM; Hill’s coefficient = 2.8).

In our earlier study, we demonstrated that constituents of nutmeg essential oil—monoterpenes sabinene and α-pinene and sesquiterpene α-copaene—were potent and efficient Cx43 GJ inhibitors [4]. In the current study, we aimed at examining the effect of another constituent of nutmeg essential oil, the monocyclic monoterpene d-limonene, on the conductance of GJs composed of Cx43 (Figure 5A,B) exogenously expressed in HeLa cells. Before experimental testing, the IC_50_ values were evaluated by molecular docking, QSAR, and 3D-QSAR models (Table 1). IC_50_ies determined by these approaches and converted to molar concentrations were 66, 14, and 42 µM, respectively.

To determine the effect of d-limonene on Cx43 GJ conductance, we performed dual whole-cell patch-clamp experiments in pairs of HeLa cells expressing exogenous Cx43-EGFP (Figure 5C), applying voltage ramps to Cell-1 and measuring junctional current in Cell-2 (Figure 5D). The threshold concentration of d-limonene for inhibition of g_j_ was 10 µM (Figure 5E). Further, applying higher concentrations (30, 50, and 100 µM) (Figure 5F–H), we found that g_j_ could be completely blocked with 100 µM of d-limonene. The *e*IC_50_ value of 30 µM was derived from the fit of the experimental points to Hill’s equation (Figure 5I). Hill’s coefficient was 2.8, suggesting more than one binding site on the Cx43 GJ channel, similar to that obtained for other terpenes in our earlier study [4].

## 4. Discussion

Cardiac remodeling, which involves structural and electrical changes in the heart, may be impacted by altered expression and localization of Cx43 GJs. Decreased expression of Cx43 proteins and a heterogeneous arrangement of channels can impair cardiac conduction and lead to supraventricular or ventricular arrhythmias [48]. Cx channels are promising pharmacological targets because inhibitors of Cx channels could be useful for treating not only arrhythmias but also other communication-dependent diseases affecting other body systems. The importance of Cx43 has been well established, particularly in the heart, where a knockout of Cx43 leads to abnormal cardiac development and death at birth [49]. Abnormalities in Cx43 organization and regulation have also been linked to myocardial ischemia [48]. So, Cx43 is a considerable drug target, especially during heart ischemia and reperfusion [14,50,51]. Unfortunately, most GJ inhibitors are non-specific compounds (see introduction). For example, the Cx43 inhibitor digoxin (analyzed in QSAR here) isolated from *Digitalis lanata* is well known in cardiology [52]. It is used to treat both irregular heartbeats [53] and heart failure [54], but its side effects like gynecomastia are also significant, which could be explained by its chemical similarity to estrogen [55]. Another Cx43 inhibitor, quinidine, is also a popular antiarrhythmic drug [56]. Another examined compound, carbenoxolone, is used for the treatment of peptic, esophageal, and oral ulceration and inflammation; however, it has also been shown in humans to slow myocardial conduction [57]. The anti-malarial drug mefloquine may lead to complete heart arrest [58] and a number of neuropsychiatric effects, including suicide [59]. More recently, Cx-inhibiting peptides (antiarrhythmic peptides AAP10; ZP123; GAP-134; RXP-E; and the Cx43 mimetic peptides Gap 26 and Gap 27) were suggested for the treatment of arrhythmias in patients with ischemic cardiomyopathy [60]. On the other hand, peptides underperformed as drug candidates due to unfavorable characteristics, mainly regarding their pharmacokinetic behavior, including plasma stability, membrane permeability, and circulation half-life [61]. The discovery of new modulators of GJ channel function lacking similarity to steroid hormones to avoid side effects is of interest to human health [14,50,51]. Additionally, a long-standing challenge in the study of GJs is the lack of specific, high-affinity activators and inhibitors of GJ channels [5,62]. Therefore, it is important to predict in silico which substances could effectively modulate GJ conductance and then experimentally examine their potency, specificity, and selectivity. This could also serve as an innovative approach to repurposing licensed drugs with predicted new GJ inhibitory properties for other communication-dependent illnesses.

In our study, the results obtained by molecular docking had a worse correlation with −log(*e*IC_50_) (R = 0.78) compared with QSAR (R = 0.88) and 3D-QSAR (R = 0.90) modeling. So, it might be concluded that QSARs outperform docking. Moderate correlation in the case of molecular docking can be explained by the discrepancy often found between the binding affinity and potency of inhibition [63]. The performance of 3D-QSAR could be enhanced by achieving a more accurate alignment of closely related inhibitor structures. Other OPEN3DQSAR applications have reported O3A_SCORE values higher than the 1502 value obtained in this study [47].

All investigated Cx43 inhibitors docked at the same common docking site, suggesting that this site should be explored further when searching for more potent inhibitors. Thus, even if molecular docking accuracy is lower, it can be used in combination with QSAR and/or 3D-QSAR. Using these approaches, the −log(*p*IC_50_) of limonene was calculated to be equal to 4.17, 4.84, and 4.35, respectively, and a −log(*e*IC_50_) value of 4.52 was later determined by our patch-clamp experiments. Therefore, this new Cx43 inhibitor could be added to the current GJ inhibitor nomenclature. Considering that cardiac conduction can be altered by changes in GJ and sodium channel properties, it would be interesting to test d-limonene on sodium channels in human cardiac myocytes. It is already known that a nutraceutical product containing d-limonene, extracted from *Cannabis sativa*, modulates voltage-gated sodium channel function [64]. The compounds that inhibit GJs with no or a small effect on sodium channels would contribute to better understanding the role of ephaptic transmission [65].

Based on our findings, we conclude that when searching for potent, selective, and specific inhibitors of GJ channels, it is essential to begin with a primary screening of the putative compounds using the QSAR model, followed by validation of the most appropriate candidates using patch-clamp techniques.

## Data Availability

All data are provided within the article or Supplementary Material.

## Supplementary Materials

The following supporting information can be downloaded at: https://www.mdpi.com/article/10.3390/biomedicines11071972/s1, Video S1: Three-dimensional version of Figure 1. Figure S1: The zoomed-in version of Figure 1, where all inhibitors are depicted separately.
