# Supplementary material for: Evaluation of Cx43 Gap Junction Inhibitors Using a Quantitative Structure-Activity Relationship Model
Source: Biomedicines. 2023 Jul 12;11(7):1972. doi: 10.3390/biomedicines11071972 (PMC10377234; doi:10.3390/biomedicines11071972)
Supplement: Supplementary file 1 [file biomedicines-11-01972-s001.zip › Supplementary Figure 1.pptx]

## Slide 1
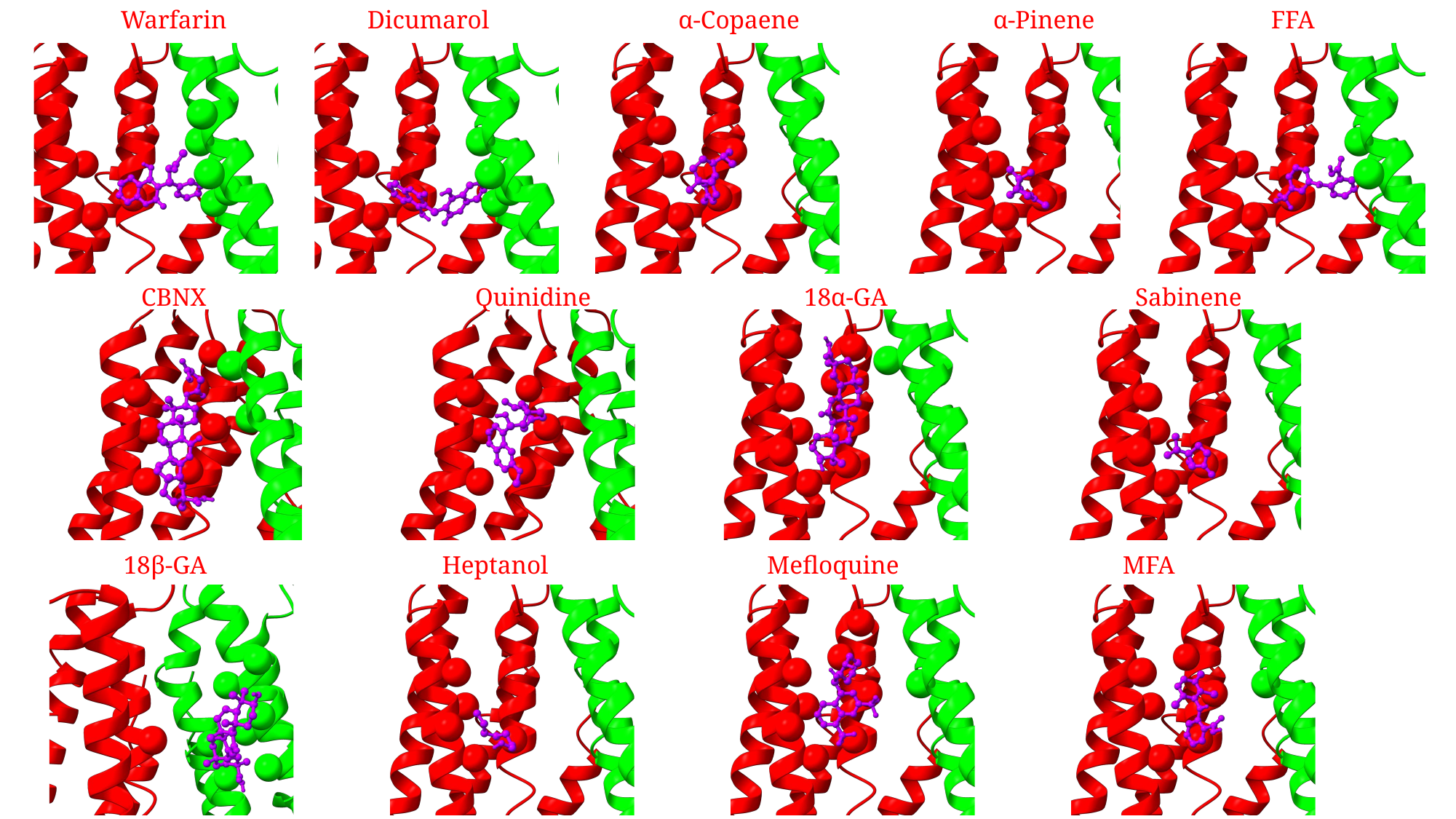

Warfarin
Dicumarol
α-Copaene
α-Pinene
FFA
CBNX
Quinidine
18α-GA
Sabinene
18β-GA
Heptanol
Mefloquine
MFA
